# Semicentennial Anniversary of Anesthesia

**Published:** 1896-11

**Authors:** 


BUFFALO MEDICAL JOURNAL.

A Monthly Review of Medicine and Surgery.

*EDITORS:*

THOMAS LOTHROP, M. D. - - WM. WARREN POTTER, M. D.

All communications, whether of a literary or business nature, should be addressed to the managing editor: 284 FRANKLIN STREET, BUFFALO, N. Y.

VOL. XXXVI. NOVEMBER, 1896. No. 4.

### SEMICENTENNIAL ANNIVERSARY OF ANESTHESIA.

ON FRIDAY evening, October 16, 1896, a meeting was held in the Alumni Hall of Buffalo University Medical College commemorative of the discovery and practical application of anesthesia to surgical operations, it being the fiftieth anniversary of that event. In response to invitations, a large number of physicians and friends of the college filled the hall at an early hour, a considerable proportion of the audience being women. Promptly at 8.15 o'clock, the Hon. James O. Putnam, chancellor of the university, appeared, accompanied by Prof. Roswell Park, Prof. E. M. Moore, of Rochester, Hon. T. Guilford Smith, regent of the University of the State of New York, and several members of the faculty of the University of Buffalo.

The exercises opened with an address by Chancellor Putnam that we print in another column of this issue of the JOURNAL. It will appear to those who were present needless for us to say that the learned chancellor was at his best on this occasion, and that his well-chosen, eloquent words fell in pleasing cadences upon the ears of his listeners. Next followed an address by Dr. Roswell Park that also appears in this edition, and which was replete with instructive history on the subject of anesthesia in general and its application to surgery in particular. We quite agree with Dr. Park in his analysis of the rights of the respective claimants to the discovery. Practically there are but four claims to be considered in this relation, and their rights have been fairly dealt with, as well as equitably summed up by the speaker of the evening. We commend a careful reading of this address to all interested in the subject.

When the venerable and erudite Professor Moore was introduced by the chancellor he was received with a storm of applause which lasted some minutes. How fittingly the words of Chancellor Putnam were chosen in presenting him: "He has been well styled," said the chancellor, "by his brethren, their Nestor. If now in his advancing years Professor Moore be like Mont Blanc, snow-capped, he is like Mont Blanc in another respect, the highest peak in his professional range."

Dr. Moore entertained the audience most delightfully with pleasant reminiscences of his surgical experiences in the pre-anesthetic period. He was rich in thought, easy in expression, entertaining in anecdote and wise in his utterances. There were many in the audience who had listened to Professor Moore when he was an honored teacher of surgery at the college, and who eagerly sought the opportunity to shake his hand in cordial good wishes after the conclusion of the ceremonies.

After Dr. Moore's speech, the chancellor called upon Drs. C. C. Wyckoff and John Hauenstein, who entertained the audience for a few minutes with local reminiscences of the early days of surgical practice, before and about the time of the introduction of anesthesia. It was an occasion long to be remembered by those present, and reflects much credit upon the faculty, who planned and carried out the commemoration of an event that perhaps is not equaled in its importance by any other that has happened in the history of surgery.

We devote considerable space in this edition of the JOURNAL to the publication of the addresses for which we need make no apology. The pictures that are introduced in connection with it cannot fail to prove of interest, and we trust that our readers will be entertained, if not instructed, by a careful examination of the record. The portrait of Dr. Morton, and the scene at the Massachusetts general hospital, we have reproduced from the Trials of a Public Benefactor.

We desire to call special attention to the proceedings of the Medical Society of the County of Erie, held in February, 1865, when Dr. Morton visited Buffalo in connection with his discovery. We think it a fitting climax to the anniversary proceedings to publish these minutes, that they may be preserved as a part of the history of the occasion, and as practically showing that Buffalo was not slow to recognise Dr. Morton's just claim to the sympathies and substantial aid of the profession and the people.

